# The Rice Cation/H^+^ Exchanger Family Involved in Cd Tolerance and Transport

**DOI:** 10.3390/ijms22158186

**Published:** 2021-07-30

**Authors:** Wenli Zou, Jingguang Chen, Lijun Meng, Dandan Chen, Haohua He, Guoyou Ye

**Affiliations:** 1 Group of Crop Genetics and Breeding, Jiangxi Agricultural University, Nanchang 330045, China; zwl@stu.jxau.edu.cn; 2 CAAS-IRRI Joint Laboratory for Genomics-Assisted Germplasm Enhancement, Agricultural Genomics Institute at Shenzhen, Chinese Academy of Agricultural Sciences, Shenzhen 518120, China; chenjg28@mail.sysu.edu.cn (J.C.); menglijun@caas.cn (L.M.); chendandan202107@163.com (D.C.); 3 Shenzhen Branch, Guangdong Laboratory of Lingnan Modern Agriculture, Genome Analysis Laboratory of the Ministry of Agriculture and Rural Affairs, Agricultural Genomics Institute at Shenzhen, Chinese Academy of Agricultural Sciences, Shenzhen 518120, China; 4 School of Agriculture, Sun Yat-sen University, Guangzhou 510275, China; 5 Rice Breeding Innovations Platform, International Rice Research Institute, Metro Manila 1301, Philippines

**Keywords:** cadmium, cadmium translocation, cadmium tolerance, cation/H^+^ exchanger, rice (*Oryza sativa* L.)

## Abstract

Cadmium (Cd), a heavy metal toxic to humans, easily accumulates in rice grains. Rice with unacceptable Cd content has become a serious food safety problem in many rice production regions due to contaminations by industrialization and inappropriate waste management. The development of rice varieties with low grain Cd content is seen as an economic and long-term solution of this problem. The cation/H^+^ exchanger (CAX) family has been shown to play important roles in Cd uptake, transport and accumulation in plants. Here, we report the characterization of the rice *CAX* family. The six rice *CAX* genes all have homologous genes in *Arabidopsis thaliana*. Phylogenetic analysis identified two subfamilies with three rice and three *Arabidopsis thaliana* genes in both of them. All rice *CAX* genes have trans-member structures. *OsCAX1a* and *OsCAX1c* were localized in the vacuolar while *OsCAX4* were localized in the plasma membrane in rice cell. The consequences of qRT-PCR analysis showed that all the six genes strongly expressed in the leaves under the different Cd treatments. Their expression in roots increased in a Cd dose-dependent manner. GUS staining assay showed that all the six rice *CAX* genes strongly expressed in roots, whereas *OsCAX1c* and *OsCAX4* also strongly expressed in rice leaves. The yeast (*Saccharomyces cerevisiae*) cells expressing *OsCAX1a*, *OsCAX1c* and *OsCAX4* grew better than those expressing the vector control on SD-Gal medium containing CdCl_2_. *OsCAX1a* and *OsCAX1c* enhanced while *OsCAX4* reduced Cd accumulation in yeast. No auto-inhibition was found for all the rice *CAX* genes. Therefore, *OsCAX1a*, *OsCAX1c* and *OsCAX4* are likely to involve in Cd uptake and translocation in rice, which need to be further validated.

## 1. Introduction

Cadmium (Cd) is one of the most toxic heavy metal elements in the environment and has an inhibitory effect on the photosynthesis rate, enzyme activity and ion absorption of plant [1,2,3,4]. Cd stress causes the overproduction of reactive oxygen species (ROS), resulting in oxidative stress and negatively affects the defense system of plants [5,6,7], which, in turn, affects the growth and development, and ultimately reduces the yield, nutritional quality and taste of rice [8]. Moreover, Cd can be easily absorbed by rice and accumulated in grains, which are the staple food for more half of the world population [9,10]. Rice with unacceptable Cd content has become a serious food safety problem in many rice production regions due to contaminations caused by industrialization and inappropriate waste management [11,12]. The development of rice varieties with low grain Cd content is seen as an economic and long-term solution of this problem [13,14,15], since rice varieties show significant genetic variation in the ability to accumulate Cd [16,17]. Identification of varieties with high tolerance to Cd and low grain Cd content is feasible. To facilitate the use of molecular breeding methods, including marker-assisted selection, genetic engineering and genome editing, it is important to identify quantitative trait loci (QTLs) and key genes for the regulation of Cd absorption, transport, distribution and accumulation. QTLs identified by using linkage mapping and association analysis in rice have been summarized by Chen et al. (2019) [18].

Cd is absorbed from soil by roots and then translocated into shoots, and finally accumulated in the grains and other tissues [19]. Mechanisms are found to regulate absorption, transportation and accumulation of Cd in plants. Therefore, many genes are involved in the determination of Cd tolerance and acumination and have been reported to play some roles in Cd regulation in rice [18]. The following gene families appear to be particularly important: the mitogen-activated protein kinase (MAPK) family [20,21,22], the cation diffusion facilitator (CDT) family [23], ATP-binding cassette transporter (ABC) superfamily [24,25,26], the ZRT/IRT-like protein (ZIP) family [27,28,29,30], the heavy metal ATPase (HMA) family [31,32,33,34,35,36], the metal tolerance protein (MTP) family [37,38] and the natural resistance-associated macrophage protein (NRAMP) family [39,40,41,42,43,44].

In recent years, the tonoplast Ca^2+^/H^+^ exchanger (CAX) family, a member of the Ca^2+^/cation antiporter superfamily [45], has drawn great interest of researchers. Studies in *Arabidopsis* and other plant species have shown that *CAX* genes play important roles in the tolerance of multi-cation, metal transport, elemental distribution and abundance, ion homeostasis and the responses to other abiotic stresses [46,47]. Transgenic lines of *CAX* genes in multiple species have been shown to be involved in Cd regulation. The *cax1* mutant in *Arabidopsis thaliana* caused higher Cd sensitivity at low concentrations of calcium (Ca), and a stronger accumulation of reactive oxygen species after Cd treatment [48,49]. *AtCAX2* and *AtCAX4* were reported to confer tolerance to high toxic levels of Cd, zinc (Zn) and manganese (Mn) in tobacco (*Nicotiana tabacum* L.) [50,51], while root-selective expression of *AtCAX4* and *AtCAX2* resulted in reduced leaf Cd in tobacco [52]. Overexpression of *TuCAX1a* and *TuCAX1b* from *Triticum urartu* could improve the tolerance and translocation to exogenous Ca and Zn, and inhibit Cd translocation in *Arabidopsis* [53]. The overexpression of *SaCAX2h* from *Sedum alfredii* enhanced the accumulation of Cd in transgenic tobacco [54]. In addition, all *Arabidopsis CAX* genes except the uncharacterized *AtCAX6* involved in low Ca tolerance and Ca transport in yeast [55,56,57]. *AtCAX5* was also involved in Mn transport and ion homeostasis in yeast [47,58]. *AtCAX2* was found to participate in Ca transport and accumulation in both tomato (*Lycopersicon esculentum* L.) and potato (*Solanum tuberosum* L.) [59]. The *Atcax1* mutant displayed tolerance to Mn and Mg (magnesium) toxicity and Ca deficiency [60] and increased *CBF*/*DREB1* expression and cold-acclimation response in *Arabidopsis* [55]. The loss-of-function of both *AtCAX3* and *AtCAX4* exhibited salt sensitivity in *Arabidopsis* [61,62]. Heterologous expression in yeast indicates that all rice *CAX* genes, except *OsCAX2*, confer tolerance to low Ca [63], and *OsCAX4* is also involved in the transport of Ca, Mn and copper (Cu) [64]. However, the roles of the rice CAX transporter family in Cd uptake and transport have not yet been explored. Therefore, this study aimed to characterize rice *CAX* genes, paying particular attention to the regulation of Cd. Bioinformatics approaches were used to conduct phylogenetic analysis and gene-structure analysis, including the number of exons and introns, and transmembrane structure. The expression pattern was studied by using qRT-PCR. Tissue-specificity of expression in the seedling stage was detected by GUS staining. Subcellular localization of the functional genes was investigated by transiently expressing GFP-gene fusion into rice protoplasts. Cd transport activity was tested by transforming yeast strains BY4741. In addition, the functions of the *CAX* family genes in ion transport in multiple species were summarized to assist in studying the roles of the rice *CAX* genes in other metals and ions. The results of this study have important theoretical significance and application value for rice genetics and breeding.

## 2. Results

### 2.1. Bioinformatics Analyses of CAX Family Genes in Oryza sativa and Arabidopsis thaliana

**Figure 1:**
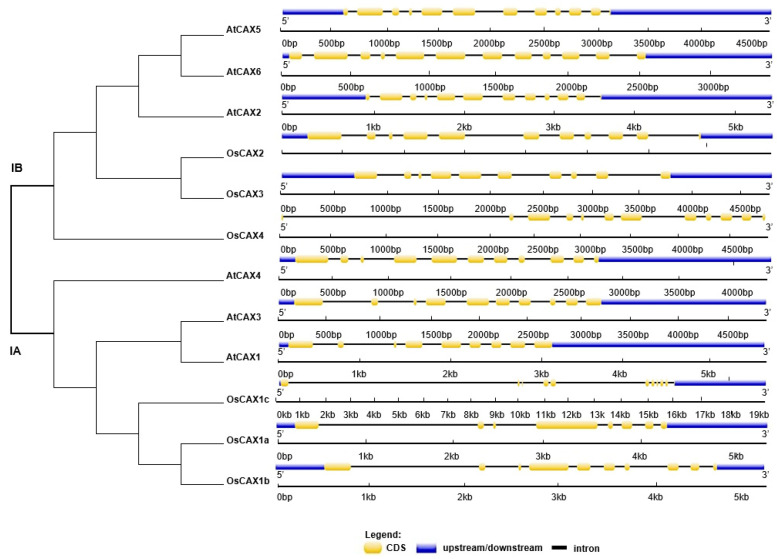
Phylogenetic relationship of *CAX* family genes of *Oryza sativa* and *Arabidopsis thaliana*. The phylogenetic tree was constructed based on sequence alignment of CAX homologs from *Arabidopsis* (At) and rice (Os), using the neighbor-joining method with bootstrapping analysis implemented in MEGA 7.0. The CAX proteins are clustered into two groups (Type IA and Type IB). Gene structures were drawn, using Gene Structure Display Server 2.0 with genomic sequences and CDS sequences. Introns and exons are represented by black lines and yellow boxes, respectively. Blue boxes represent UTRs.

Both *Arabidopsis* and rice have six *CAX* genes. All identified *CAX* members were named based on their phylogenetic relationships (Figure 1). The two subfamilies (Type IA and Type IB) formed by the two main branches of the phylogenetic tree each have three genes from *Arabidopsis* and three genes from rice (Figure 1). Chromosome mapping showed that the six rice *CAX* genes are distributed on chromosomes 1, 2 (two genes), 3, 4 and 5 (Figure 1 and Table 1). The six *CAX* genes of *Arabidopsis* are distributed on chromosomes 1 (two genes), 2, 3 (two genes) and 5 (Figure 1 and Table 1). The CDS regions of the rice *CAX* genes range in length from 1089 to 1362 bp and encoded proteins with lengths of 363–454 amino acid residues, molecular weights of 39.07–49.11 KDa and *pI* values of 4.57–7.00 (Table 1). The length of CDS, encoded proteins with lengths, molecular weights and *pI* values of the *Arabidopsis CAX* genes range in length from 1326 to 1428 bp, 441–475 amino acid residues, 48.10–51.84 KDa and *pI* values of 4.45–6.51, respectively (Table 1). *AtCAX1*, with a relatively long length, appears to be distinct from the other five members of the Subfamily IA proteins (Table 1). *OsCAX4*, with a relatively short length, appears to be divergent from the other five members of the Subfamily IB proteins (Table 1). Gene structures were different in each of the two main subfamilies, as illustrated in Figure 1. Subfamily IA members have 8–11 exons and 7–10 introns. Subfamily IB members have 10–12 exons and 9–11 introns. The four members of the IB subfamily (*AtCAX2*, *AtCAX5*, *AtCAX6* and *OsCAX4*) all have 12 exons and 11 introns. In particular, *OsCAX4* was classified into Type IB phylogenetically and has no UTR (Figure 1). The rice CAX proteins have 8–11 putative transmembrane domains (TMDs) (Table 1 and Figure S1). Except for *AtCAX3*, which contains 11 TDMs, all other *Arabidopsis* CAX proteins have 10 putative TDMs (Table 1 and Figure S1).

In BLASTP analysis, the identity between the *CAX* genes is high with a minimum value of 54%, indicating that they are relatively conservative (Figure S2a). The Subfamily IB members have a higher degree of similarities, reaching 71% (Figure S2b), in comparison to the Subfamily IA members, which have their highest identity as 64% (Figure S2c). Sequence identity in aligned regions ranges from 31 to 88% outside of selfhits (Table 2), with the highest percentage of identity being between AtCAX5 and AtCAX6. The lowest identity is between OsCAX1c and OsCAX4 (Table 2). These results indicated that the *CAX* gene family has high homology in *Oryza sativa* and *Arabidopsis thaliana*.

### 2.2. Response of Rice CAX Family Genes to Cd Stress

**Figure 2:**
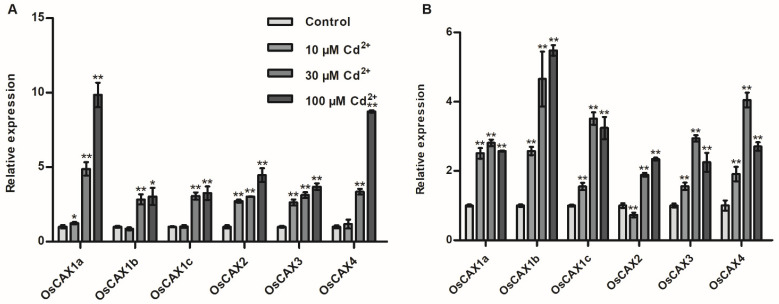
Expression of rice *CAX* genes under CdCl_2_ treatments. The relative transcript levels were quantified by qRT–PCR in (**A**) roots and (**B**) leaves (mixed collection of shoots and leaves) of rice variety Nipponbare grown in hydroponic culture under 0, 10, 30 or 100 μM CdCl_2_ treatment with the IRRI solution for 7 days. The rice actin gene (*Rac1*) was used as an internal reference to normalize gene-expression data. Statistical comparison was performed by one-side *t*-test (* *p* < 0.05 and ** *p* < 0.01). Values are mean ± SE (*n* = 3, three replicates with tissues being collected from three plants per replicate, three plants per pool).

The *CAX* genes of *Arabidopsis* and a few other species to Cd stress have been widely reported [58,65]. We analyzed the expression profiles of all the six rice *CAX* family genes, using qRT-PCR, under different CdCl_2_ treatments. The transcription levels of all the six genes were significantly upregulated in roots under the 30 and 100 μM CdCl_2_ treatments, and the expressions were increased with increasing Cd concentrations (Figure 2A). The highest expression was observed for *OsCAX1a* in roots treated with 100 μM CdCl_2_ (Figure 2A). Under 10 μM CdCl_2_ treatment, the expression of *OsCAX1b*, *OsCAX1c* and *OsCAX4* in roots was similar to the untreated control (Figure 2A). The transcription levels in leaves were also significantly induced by all Cd treatments (Figure 2B). *OsCAX1b* and *OsCAX2* showed relatively high expression in leaves under 100 μM CdCl_2_, with *OsCAX1b* being the highest among the six genes, while the other four *CAX* genes were highly expressed in leaves when treated at 30 μM CdCl_2_ (Figure 2B). These results suggested that the rice *CAX* genes could respond to Cd stress.

### 2.3. Functional Analysis of Rice CAX Genes in Yeast

**Figure 3:**
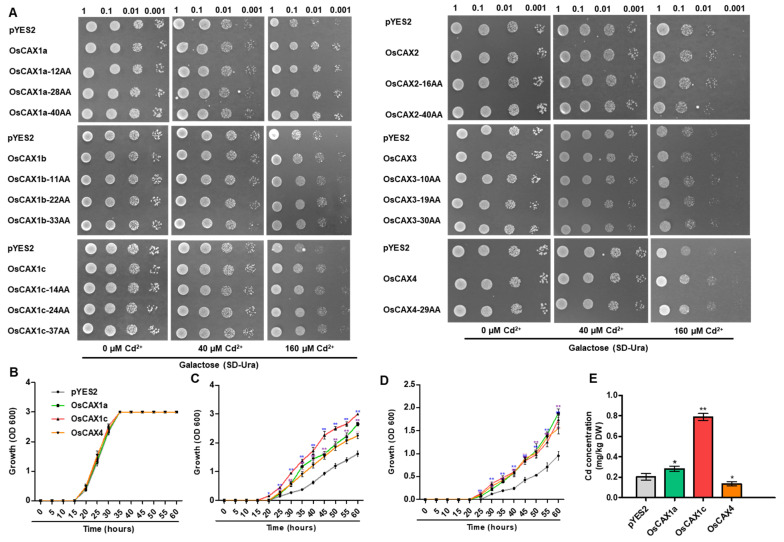
Functional assay of rice *CAX* genes by heterologous expression in yeast. (**A**) Yeast mutant strain expressing the empty vector pYES2 or vectors for *CAX* genes grown in SD-Ura medium containing galactose without Cd or with different Cd concentrations. Pictures were taken after 2 days of growth at 30 °C. The growth curve of empty vector, *OsCAX1a*, *OsCAX1c* and *OsCAX4* transformed yeast BY4741 strain in liquid medium with (**B**) 0, (**C**) 20 or (**D**) 40 μM CdCl_2_. The absorbance at 600 nm (OD600) of cell cultures was measured every 5 h. (**E**) Cd accumulation in the wild-type BY4741 (gray) and BY4741 expressing *OsCAX1a* (green), *OsCAX1C* (red) and *OsCAX4* (orange) treated with 5 μM CdCl_2_ for 24 h. Statistical comparison was performed by one-side *t*-test (* *p* < 0.05 and ** *p* < 0.01). Error bar indicates standard deviation, and the data are presented as means ± SD (*n* = 3).

Since the phenomena of auto-inhibition at the N-terminus have been reported for *CAX* genes in multiple species [58,66,67,68,69], the full-length rice *CAX* genes and their 15 N-terminal truncated versions were included in our functional test by using heterologous yeast assay. To create the truncated version without affecting the transmembrane structure, we removed several amino acids from the N-terminal (Figure S4). The truncated versions used were three *ΔOsCAX1a* (*OsCAX1a-12AA*, *OsCAX1a-28AA* and *OsCAX1a-40AA*), three *ΔOsCAX1b* (*OsCAX1b-11AA*, *OsCAX1b-22AA* and *OsCAX1b-33AA*), three *ΔOsCAX1c* (*OsCAX1c-14AA*, *OsCAX1c-24AA* and *OsCAX1c-37AA*), two *ΔOsCAX2* (*OsCAX2-16AA* and *OsCAX2-40AA*), three *ΔOsCAX3* (*OsCAX3-10AA*, *OsCAX3-19AA* and *OsCAX3-40AA*) and one *ΔOsCAX4* (*OsCAX4-29AA*). Then they were expressed in wild-type yeast strain BY4741(MATa his3Δ1 leu2Δ0 met15Δ0 ura3Δ0). On a SD-Gal medium without Cd, there were no differences in growth between the control carrying an empty vector (vector control) and the *CAX*-carrying yeast strains (Figure 3A). For the truncated versions of genes, the yeast cells expressing *OsCAX1a, OsCAX1c, OsCAX4* had better growth than the vector control on SD-Gal medium containing 160 μmol/L CdCl_2_, while the other three genes showed no significant difference with the vector control on the SD-Gal medium containing 40 or 160 μmol/L CdCl_2_ (Figure 3A). Consequently, we did not find N-terminal auto-inhibition of the rice *CAX* genes.

Further experiments using liquid media with different Cd concentrations were conducted to confirm their Cd transport activity. Without Cd, all *CAX* genes expressing yeast cells showed similar growth to the control. However, the growth of BY4741 was significantly promoted by *OsCAX1a* (*ΔOsCAX1a*), *OsCAX1c* (*ΔOsCAX1c*) and *OsCAX4* (*ΔOsCAX4*) when 10, 20 or 40 μmol/L CdCl_2_ was supplemented (Figure 3B–D and Figure S3). In addition, N-terminal auto-inhibition was still not observed in experiments using liquid medium either (Figure S3). Under the 24 h Cd exposure, *OsCAX1a* and *OsCAX1c* enhanced Cd accumulation, while *OsCAX4* reduced Cd accumulation (Figure 3E). These results indicated that *OsCAX1a*, *OsCAX1c* and *OsCAX4* had Cd transport activity in yeasts and might be involved in Cd uptake, transport and accumulation in rice.

### 2.4. Expression Pattern and Subcellular Localization of the Rice CAX Genes

**Figure 4:**
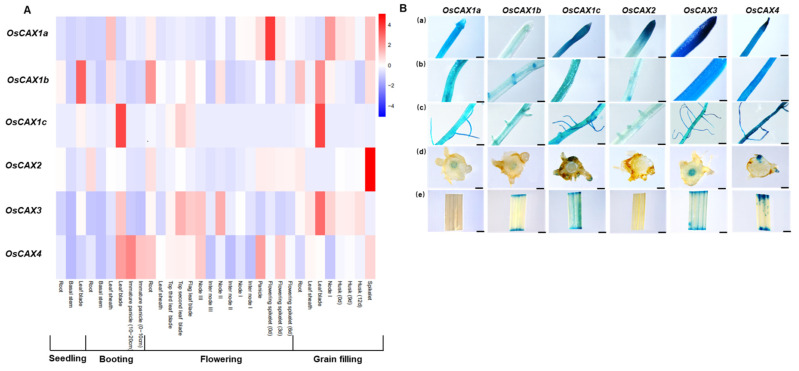
Tissue expression pattern. (**A**) Relative expression of rice *CAX* members in various tissues at different growth stages. Various tissues samples were taken from cv. Nipponbare grown in a paddy field. (**B**) Localization of *pOsCAXs-GUS* expression in transgenic rice plants. GUS staining pattern in tissues of 14-day-old seedlings cultured with de-ionized water: (**a**) root tip, (**b**) 2 to 3 cm from the root tip, (**c**) root lateral branching zones, (**d**) hand-cut cross-section of the root–shoot junction and (**e**) leaves. (**a**–**c**) Scale bar = 0.5 mm. (**d**,**e**) Scale bar = 2 mm.

To investigate the expression patterns of rice *CAX* genes, the expression in tissues of seedlings, booting, flowering and grain filling stages grown under normal field conditions were measured by qRT-PCR. We found that *OsCAX1c* was specifically expressed in leaves, with very limited expression elsewhere, while other genes were detected in all tissues of different growth stages (Figure 4A). It was obvious that *OsCAX1c*, *OsCAX3* and *OsCAX4* were strongly expressed in leaves (Figure 4A). Both *OsCAX1a* and *OsCAX2* were expressed at a high level in flowering spikelet but weakly in leaves. The expression of *OsCAX1b* in roots and leaves was slightly higher than in other tissues (Figure 4A).

To further investigate their tissue-specific expression, we developed stable transgenic rice lines expressing the GUS reporter gene driven by the *OsCAX1a*, *OsCAX1b*, *OsCAX1c*, *OsCAX2*, *OsCAX3* or *OsCAX4* (−2000 bp from the start codon) promoter. Transgenic seedlings were cultured in deionized water for 14 days, and then various tissues were collected and stained for GUS detection (Figure 4B). All the six genes were observed in roots including the root tip (0.2 cm from the root tip), root hair (2 to 3 cm from the root tip) and root lateral branching zones. *OsCAX3* and *OsCAX4* expressed abundantly throughout the primary and lateral roots. *OsCAX3* strongly expressed throughout the stele in primary roots, whereas other genes weakly expressed or hardly expressed at the base of the stele (Figure 4B). All genes, except *OsCAX1b*, expressed in the primary root tip. In the root–shoot junction, *OsCAX1a*, *OsCAX1b* and *OsCAX3* strongly expressed, with *OsCAX3* showing slightly higher expression than *OsCAX1a* and *OsCAX1b*. *OsCAX1a* and *OsCAX2* hardly expressed in leaves; *OsCAX1c*, *OsCAX3* and *OsCAX4* were more strongly expressed in leaves than *OsCAX1b* (Figure 4B). These results were consistent with the (qRT–qPCR) results shown in Figure 4A.

**Figure 5:**
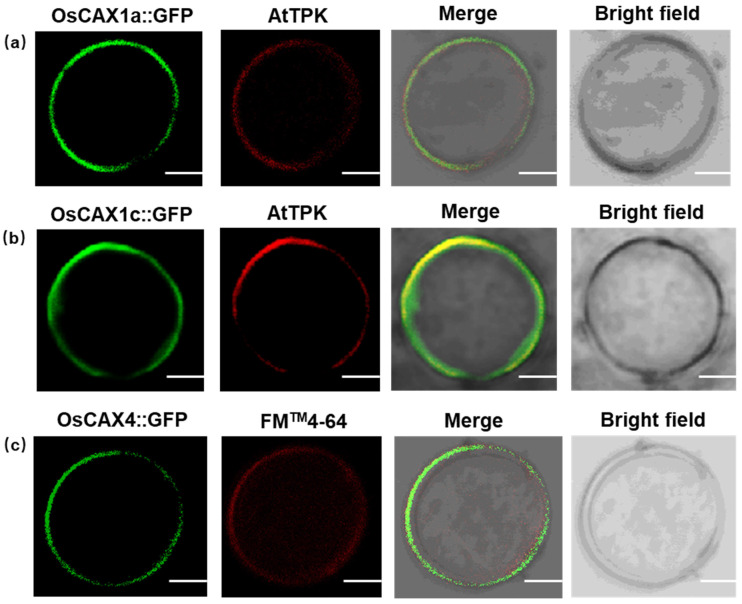
Subcellular location of *OsCAX1a*, *OsCAX1c* and *OsCAX4* in rice protoplasts. (**a**) The fluorescence of OsCAX1a-GFP, AtTPK (**left**), and overlay of FM4–64 and OsCAX1a-GFP, bright field (**right**) are shown, respectively. (**b**) The fluorescence of OsCAX1c-GFP, AtTPK (**left**), and overlay of AtTPK and OsCAX1c-GFP, bright field (**right**) are shown, respectively. (**c**) The fluorescence of OsCAX4-GFP, FM^TM^4–64 (**left**), and overlay of FM^TM^4–64 and OsCAX4-GFP, bright field (**right**) are shown, respectively. Bars = 10 μm.

To determine the subcellular localization of *OsCAX1a*, *OsCAX1c* and *OsCAX4* proteins in plant cells, we constructed GFP fusion protein and transformed it into rice-sheath protoplasts. OsCAX1a, OsCAX1c and OsCAX4 fused with GFP was observed at the periphery protoplasts (Figure 5). Moreover, *OsCAX1a* and *OsCAX1c* were co-localized with the tonoplast membrane marker AtTPK, suggesting that both *OsCAX1a* and *OsCAX1c* were localized in the vacuolar membrane (Figure 5). The merge images of OsCAX4-GFP and FM^TM^4–64 indicated the plasma membrane subcellular localization of *OsCAX4* (Figure 5). Vacuolar localization of *OsCAX1a* has been reported previously by Kamiya et al. (2005) [63]. The membrane localizations of *OsCAX1a*, *OsCAX1c* and *OsCAX4* were consistent with their cation/H^+^ exchanger functions, which suggested that the rice *CAX* genes might play important roles in Cd regulation.

## 3. Discussion

### 3.1. The CAX Family Genes in Rice Have High Identity to Their Homologues in Arabidopsis Thaliana

It is well-known that the homeostasis of intracellular Ca^2+^ plays an important role in signal transduction of stresses and is necessary for normal cell growth [44]. Hence, control of Ca^2+^ concentration is critical to cellular function. The Ca^2+^/cation antiporter (CaCA) superfamily members, including MHX (Mg^2+^/H^+^ exchanger), CCX (cation/Ca^2+^ exchanger) and CAX, are the most important Ca^2+^ transporters. The CaCA exchangers family structure is highly conserved in related plants [70]. Here, we explored the structural characteristics of CAX transporters through phylogenetic tree analysis, transmembrane structure and homology identification of CAX family proteins in rice and *Arabidopsis*. It was found that the two subfamilies formed by the two main branches of the phylogenetic tree each have three *Arabidopsis* genes and three rice genes (Figure 1), and the length of the CDS region and the encoded proteins, the molecular weight and the range of *pI* values were all relatively close in rice and *Arabidopsis* (Table 1). The gene structures of the two main subfamilies are slightly different. The members of Subfamily IA have 8–11 exons and 7–10 introns. The members of Subfamily IB have 10–12 exons and 9–11 introns, which were one more than those of the Subfamily IA members (Figure 1). CAX family proteins have 8–11 putative transmembrane domains (Figure S1). It is worth noting that the identity between *CAX* genes is high, the sequence identity in the aligned region ranges from 31 to 88% outside of selfhits (Table 2) and the homology among all *CAX* genes reached 54% (Mate Figure S2). Taken together, the *CAX* family genes in rice have high identity to their homologues in *Arabidopsis thaliana,* indicating that functional similarities are expectable, which is advantageous in leveraging the relatively more abundant information accumulated in *Arabidopsis* to speed up studies in rice.

### 3.2. N-Terminal Auto-Inhibition Was Not Found for the Rice CAX Genes Tested Using Cd Treatment

N-terminal auto-inhibition has been widely reported for *CAX* genes in different species [58,67,68,69], and it is most widely studied in *Arabidopsis* [66,71,72]. For instance, we found that deleting 36 amino acids at the N-terminus of *AtCAX1* ORF improved Ca and Mn tolerance compared to the full-length *AtCAX1* ORF by heterologous transformation of yeast [71,72]. The modified *AtCAX2* (*sCAX2A*) with a domain of *AtCAX2* and without the N-terminal autoinhibitory domain elevated Ca accumulation in the fruits of tomato. Mn transport in yeast was controlled by the N-terminus of tomato *LeCAX2* [58]. The absence of N-terminal regulatory region (NRR) of the cotton (*Gossypium hirsutum*) *GhCAX3* altered cold tolerance compared to full-length of *GhCAX3* [68]. It has also been reported in rice that the N-terminus of *CAX* family genes (Type IA) regulates Ca tolerance and transport in yeast. *OsCAX1a∆27* and *OsCAX1c∆47* strongly enhanced the Ca tolerance of yeast, and *OsCAX1b* was more sensitive to 100 mM CaCl_2_ than *OsCAX1b∆36* was [63]. We transformed the yeast strain BY4741 with all members of the rice *CAX* genes and their corresponding truncated versions with the removal of the 10–40 amino acids from the N-terminal. The Cd transport activity in yeast did not show any differences, indicating that the N-terminal auto-inhibition was not shown for Cd in rice. However, it is best not to interpret this as an indication that the N-terminal auto-inhibition is not present in the rice *CAX* genes, since we only tested for Cd. Kamiya et al. (2005) [63] reported that *OsCAX2* and the N-terminal truncation mutants of *OsCAX2* (*OsCAX2∆26*) expressed in yeast did not show Ca tolerance. The modified *Arabidopsis* CAX2 (*sCAX2A*) also showed almost no changes in Mn, Cu and Fe accumulation in the fruits, compared to the overexpression of *AtCAX2* in tomato [73]. More detailed studies are needed to (dis)validate this observation.

### 3.3. Rice CAX Genes, Particularly OsCAX1a, OsCAX1c and OsCAX4, Might Be Important in Controlling Cd Uptake and Translocation in Rice

**Figure 6:**
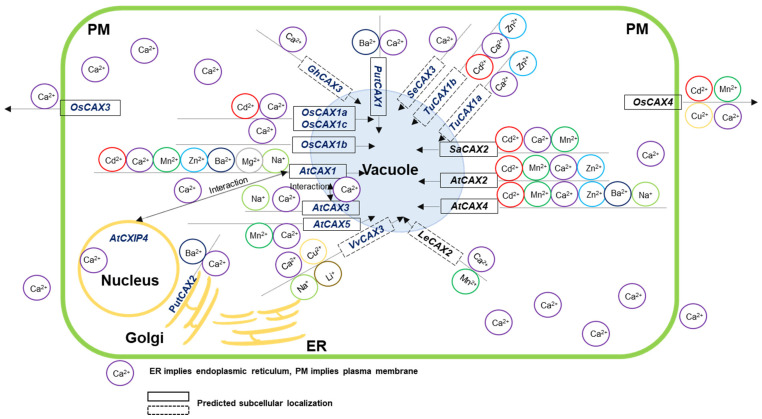
Model of the involvement of *CAX* family members in ion transport in plants. Ions are transported with the help of *CAX* genes. The solid black border indicates the subcellular location of the known genes, and the dotted line indicates the subcellular location predicted by CELLO v2.5 (accessed on 21 May 2021; http://cello.life.nctu.edu.tw/).

Existing studies have shown that the *CAX* genes in plants are involved in ion tolerance, transport and homeostasis, as shown in Figure 6 and Table S3. However, the response of rice *CAX* genes to Cd has not been studied. Our results showed that the expression of all the six rice *CAX* genes were upregulated by high-concentration CdCl_2_ treatments. This was particularly true in roots, for which gene expression increased with increasing Cd concentration (Figure 2). By transforming the BY4741 yeast strain, we found that *OsCAX1a*, *OsCAX1c* and *OsCAX4* had better Cd tolerance than the control on a SD-Gal medium containing 160 μmol/L CdCl_2_ and more active in liquid media containing Cd (Figure 3A–D). *OsCAX1a* and *OsCAX1c* increased, while *OsCAX4* decreased, the accumulation of Cd in yeast (Figure 3E). Thus, *OsCAX1a*, *OsCAX1c* and *OsCAX4* involve in Cd tolerance and transport in yeast, implying that they might involve in Cd tolerance and accumulation in rice as well. As discussed above, the rice *CAX* family genes have high identity to their homologous genes in *Arabidopsis*, indicating that their functions may be conserved. Three *Arabidopsis CAX* genes, namely *AtCAX1* [48,49], *AtCAX2* and *AtCAX4* [50,51,52], were verified by transgenic methods to participate in Cd tolerance, transport and accumulation (Figure 6 and Table S3). *OsCAX1a* has very high homology with *AtCAX1* and *AtCAX4*, reaching 64% and 56%, respectively (Figure 1 and Table 2)*. OsCAX1c* has high homology with *AtCAX1* (57%) and *AtCAX4* (49%) (Figure 1 and Table 2). *OsCAX1a* strongly expressed in spikelet and nodes, while *OsCAX1c* very strongly expressed in leaves (Figure 4A). Both of *OsCAX1a* and *OsCAX1c* are most likely to locate on the vacuolar membrane. *OsCAX4* on plasma membrane has the highest identity (54%) to *AtCAX2* (Figure 1 and Table 2) and is strongly expressed in roots and grains. Taken together, we speculate that *OsCAX1a*, *OsCAX1c* and *OsCAX4* may play different roles in the key processes, including the absorption by the roots, the xylem loading, the distribution and transportation via the nodes, and the redistribution in the leaf via phloem. These processes collectively determine the plant tolerance and accumulation of Cd.

Although *OsCAX1b*, *OsCAX2* and *OsCAX3* did not show Cd transport activity in our yeast assay (Figure 3), their roles in Cd regulation could not be completely ruled out. Firstly, their expression levels in roots and leaves were all induced by Cd treatments (Figure 2). Secondly, *OsCAX1b* and *OsCAX3* were previously reported to involve in tolerance to Ca, and *OsCAX3* also confers Mn tolerance in yeast [63]. Thirdly, CAX proteins function as Ca^2+^/H^+^ and/or heavy metal/H^+^ exchangers [56,73]. The importance of Ca^2+^ acts as a secondary messenger in the signal transduction of various biotic and abiotic stimuli in plants has been well documented [74,75,76]. Changes in apoplastic pH or Ca^2+^ concentrations can be expected to strongly affect Cd binding capacity, since Cd^2+^ and Ca^2+^ have similar ionic radii [77]. Fourthly, *CAX* genes interact with other genes to form regulatory network controlling tolerance to metals. Yeast two-hybrid analysis showed that *AtCAX1* directly interacts with *AtSOS2*, which is a serine/threonine kinase whose function is essential for salt tolerance in *Arabidopsis* [78]. Co-expression of *AtSOS2* specifically activated *AtCAX1* and integrated Ca transport in yeast and salt tolerance in *Arabidopsis* [79]. Therefore, it is likely that *CAX* genes interact with key genes for Cd absorption and accumulation, or are regulated by transcription factors or phosphorylated by receptor kinases to affect Cd transport. Fifthly, since *CAX* genes have high degree of genetic identity, it is likely that some of the genes may have functional redundancy (Table 2). Thus, any test/assay using a single gene, as we performed, may fail to identify the function of a gene. Indeed, it has been reported that the *AtCAX1* and *AtCAX3* function as dimers, and co-expressing both *AtCAX1* and *AtCAX3* mediated Ca, lithium (Li) and salt tolerance in yeast [61,80], and affected element distribution and abundant *Arabidopsis* seeds [81].

In conclusion, we investigated all rice *CAX* genes and demonstrated that *OsCAX1a*, *OsCAX1c* and *OsCAX4* were associated with Cd tolerance and Cd transport in yeast. They are also highly likely to involve in Cd tolerance, absorption and accumulation in rice; however, further validation through transgenic methods in rice is needed.

## 4. Materials and Method

### 4.1. Identification and Bioinformatics Analyses of the Cation/H^+^ Antiporters from Oryza sativa and Arabidopsis thaliana Species

To identify CAX homologs in rice, the nucleic acid and amino acid sequences of all reported *CAX* genes in *Arabidopsis* and rice were downloaded from the rice (accessed on 16 December 2019; http://www.ricedata.cn/gene/) and *Arabidopsis* (accessed on 16 December 2019; https://www.arabidopsis.org/) databases. *CAX* genes were named according to the phylogenetic relationship between proteins. The protein molecular weight and theoretical isoelectric point (*pI*) value were calculated by ProtParam (accessed on 16 December 2019; http://web.expasy.org/protparam/). The DNAMAN software (accessed on 1 March 2019; https://www.lynnon.com/dnaman.html) was used to determine the sequence identity between rice and *Arabidopsis* CAX proteins, and the homology between subfamilies. The transmembrane structures in the proteins were predicted through TMHMM Server v2.0 (accessed on 3 June 2021; http://www.cbs.dtu.dk/services/TMHMM/). The subcellular localization of unreported CAX proteins were predicted by using CELLO v2.5 (http://cello.life.nctu.edu.tw/ (accessed on 21 May 2021; http://cello.life.nctu.edu.tw/). Multi-sequence alignment was performed with Clustal W (accessed on 1 January 2021; Clustal Omega, ClustalW and ClustalX Multiple Sequence Alignment) and drawn in Genedoc. Phylogenetic trees based on full-length protein-sequence alignments were constructed by the neighbor-joining method with 1000 bootstrap replicates, using MEGA 7.0 software (accessed on 1 January 2021; http://www.megasoftware.net/download_form). The downloaded coding DNA sequences (CDS) and genome sequences of *CAX* genes were used to construct gene structure by the Gene Structure Display Server 2.0 (accessed on 16 December 2019; http://gsds.cbi.pku.edu.cn/index.php).

### 4.2. Plant Materials and Growth Conditions

Rice (*Oryza sativa* L. cv. Nipponbare) seeds were surface-sterilized with 10% (*v*/*v*) hydrogen peroxide solution for 30 min, thoroughly rinsed, washed six times with deionized water and germinated for 48 h under dark conditions and a temperature of 30 °C [82]. The dew-white and uniformly growing seeds were sown in a 96-well polymerase chain reaction (PCR) plate (8 × 12) with perforated wells at the bottom to facilitate the roots to fully contact with the nutrient solution [83]. The nutrient solution was prepared according to the composition of IRRI solution: 1.0 mM MgSO_4_·7H_2_O, 1.25 mM NH_4_NO_3_, 0.3 mM KH_2_PO_4_, 1.0 mM CaCl_2_, 0.35 mM K_2_SO_4_, 0.5 mM Na_2_SiO_3_, 20.0 μM Fe-EDTA, 20.0 μM H_3_BO_3_, 9.0 μM MnCl_2_, 0.77 μM ZnSO_4_, 0.32 μM CuSO_4_ and 0.39 μM (NH_4_)_6_Mo_7_O_24_, at pH 5.5. The nutrient solution was replaced every three days. Rice seedlings were grown for 10 days.

The seedlings that grew consistently were moved to a rectangle 3.5 L box containing 2 L of normal nutrient solution, with a 3 × 8 foam board being used as bed, and grown for a week. Then the seedlings were grown under treatment conditions of 0, 10, 30 or 100 μM CdCl_2_. The nutrient solution was changed every two days and the night before sampling. Culture was conducted in a greenhouse, under natural light, at day/night temperatures of 30 °C/22 °C and 60% relative humidity. After a 2-week treatment, root and leave samples were rapidly taken, frozen in liquid nitrogen and stored in a refrigerator, at −80 °C, for RNA extraction and real-time quantitative reverse-transcription PCR (qRT-PCR) analysis.

### 4.3. RNA Extraction and Real-Time PCR

Total RNA was extracted by using the TRIzol reagent (Vazyme Biotech Co. Ltd., Nanjing, China). DNaseI-treated total RNAs were subjected to reverse transcription (RT) with the HiScript II Q Select RT SuperMix for qPCR (+gDNA wiper) kit (Vazyme Biotech Co. Ltd., Nanjing, China). Transcript levels of selected genes were measured by qRT-PCR, using the CFX96^®^ Real-Time PCR System using the 2 × T5 Fast qPCR Mix (SYBRGreenI) kit (Vazyme Biotech Co. Ltd., Nanjing, China). Rice actin gene (*Rac1*) was used for normalization. The ΔΔCt method was used to calculate the relative transcript abundance [78]. The primers for qRT-PCR are given in Table S1.

### 4.4. Functional Analysis of Rice CAX Genes in Yeast

The heterologous yeast assay was utilized to identify the Cd transport ability of rice CAX proteins by transforming the BY4741 yeast strain (MATa his3Δ1 leu2Δ0 met15Δ0 ura3Δ0). All *CAX* genes were amplified from full-length cDNA clones, using the PCR primers lying downstream from the start codon and just upstream of the stop codon (Table S2). The protocol for removing some amino acids from the N-terminus of the CAX transporters was described in detail by Shigaki et al. (2010) [72]. Using BamH I and EcoR I, we ligated each rice *CAX* gene into the pYES2 vector (Invitrogen), with correct direction, using a ClonExpress^®^ Ultra One Step Cloning Kit (Vazyme Biotech Co. Ltd., Nanjing, China), resulting in the pYES2-OsCAXs construct. The vectors truncated were verified by Sanger sequencing. Empty vector pYES2 and the *CAX* gene vectors were then transformed into BY4741 yeast cells, respectively, according to the manufacturer’s protocol (Yeast Transformation Kit; Beijing Coolaber Technology Co. Ltd., Beijing, China). Transformants were selected on synthetic dextrose medium without uracil (SD-Ura) and verified by PCR with yeast plasmid extraction (Yeast Plasmid Extraction Kit; Beijing Solarbio Technology Co. Ltd., Beijing, China). Positive clones were cultured on an SD-Ura liquid medium until the early logarithmic phase. For plate growth tests, yeast transformants were diluted to an OD600 of 1.0, 0.1, 0.01 and 0.001, step by step, with sterile water; 6 μL of the cell suspension was spotted on SD-Ura plates containing 0, 40 or 160 μmol/L CdCl_2_, respectively. The plates were incubated at 30 °C for 2 days before the growth phenotypes were evaluated.

To quantify the growth of BY4741 yeast strain transformed with various plasmids in liquid SD-Ura media containing CdCl_2_ overnight yeast cells were prepared and the optical density (OD) at 600 nm was adjusted to 0.8 with sterile distilled water. Then 20 μL of cell suspensions was added to 20 mL liquid SD-Ura media containing 0, 10, 20 or 40 μmol/L CdCl_2_. The OD values at 600 nm were determined at the indicated time.

For the Cd concentration determination, the yeast transformants were cultured overnight with a liquid SD-Ura medium with 2% galactose, at 30 °C and 200 rpm, until the *OD600 reached 0.8*. Then the yeast transformants were treated with 5 μM CdCl_2_ for 24 h. The yeast strain carrying empty vector was used as control. The cultures of each set were harvested in pre-weighed microfuge tubes by centrifugation and washed with sterile water for three times. After aspirating the supernatant, pelleted cells were dried in the oven, overnight, at 60 °C. The samples were wet-digested by using 5 mL concentrated HNO_3_+HClO_4_ (4 + 1), at 120 °C, in a heating block for 30 min. After cooling, the digested solution was diluted to 15 mL with deionized water. The Cd concentrations were determined by inductively coupled plasma–mass spectrometry (ICP–MS). All the assays were performed at least three times.

### 4.5. Tissue Expression Assay

For histochemical analysis of GUS activity, the upstream 2.0 kb genomic fragment of each gene was cloned into pCAMBIA1300 to generate OsCAXs _promoter_: GUS vector. The vectors were then transformed into Nipponbare to produce transgenetic plants. The GUS staining Solution (1 mg/mL) from (Beijing Coolaber Technology Co., Ltd., Beijing, China) was used to determine the activity of GUS. The samples were incubated at 37 °C for 24 h, and the solution containing (X-gluc dry powder + X-gluc solution): GUS staining buffer = 1:50. After staining, green tissue materials (such as leaves) were treated 2 or 3 times by 75% ethanol to remove chlorophyll and decolorize until the negative control material was white. GUS activity was detected by a stereoscopic fluorescence microscopy (TL5000; Leica Microsystems).

### 4.6. Subcellular-Localization Assay

Subcellular localization was investigated by transiently expressing *GFP-OsCAXs* fusion into rice protoplasts. The ORF of *OsCAX1a*, *OsCAX1c* and *OsCAX4* were amplified by PCR from rice (‘Nipponbare’) root cDNA, using primers (Table S2). Protoplasts generated from the young stems of 3-week-old rice seedlings grown under light were transformed with transient expression plasmids, according to Bart et al. (2006) [84]. GFP fluorescence signals were detected with a TCS SP5 confocal laser scanning microscope (Leica Microsystems) at 500–535 nm, after excitation at 488 nm, while FM^TM^4–64 (plasma membrane specific localization dye) was excited at 543 nm and scanned at 600–630 nm. Double staining using AtTPK (red signal) as the tonoplast membrane marker was used for further confirmation of the subcellular localization. For co-localization experiments, sequential scanning was done for both of the channels and then merged together to show overlapping signals. All the images were further processed by using Leica LAS AF Lite software.

## Figures and Tables

**Table 1:** Basic characteristics of *CAX* family genes in *Oryza sativa* and *Arabidopsis thaliana*.

Gene	Locus	Length of CDS (bp)	No. of Amino Acids (aa)	Chromosome	MW (KDa)	*pI*	Type	No. of Transmembrane Domains
*OsCAX2*	*LOC_Os03g27960*	1317	439	3	47.48	4.57	IB	10
*OsCAX3*	*LOC_Os04g55940*	1254	418	4	45.45	4.76	IB	11
*OsCAX4*	*LOC_Os02g04630*	1089	363	2	39.07	7.00	IB	8
*OsCAX1a*	*LOC_Os01g37690*	1356	452	1	47.71	6.78	IA	10
*OsCAX1b*	*LOC_Os05g51610*	1362	454	5	49.11	6.34	IA	10
*OsCAX1c*	*LOC_Os02g21009*	1353	451	2	48.16	5.62	IA	8
*AtCAX1*	*AT2G38170*	1428	475	2	51.64	6.25	IA	10
*AtCAX2*	*AT3G13320*	1326	441	3	48.21	4.45	IB	10
*AtCAX3*	*AT3G51860*	1380	459	3	49.85	5.39	IA	11
*AtCAX4*	*AT5G01490*	1365	454	5	49.61	6.51	IA	10
*AtCAX5*	*AT1G55730*	1326	441	1	48.10	4.61	IB	10
*AtCAX6*	*AT1G55720*	1404	467	1	51.84	5.40	IB	10

**Table 2:** Percentage of protein sequence identity among the CAX family proteins of *Oryza sativa* and *Arabidopsis thaliana*.

% Identity	OsCAX1a	OsCAX1b	OsCAX1c	OsCAX2	OsCAX3	OsCAX4	AtCAX1	AtCAX2	AtCAX3	AtCAX4	AtCAX5	AtCAX6
	%	%	%	%	%	%	%	%	%	%	%	%
OsCAX1a	100											
OsCAX1b	69	100										
OsCAX1c	59	58	100									
OsCAX2	50	52	43	100								
OsCAX3	49	51	40	74	100							
OsCAX4	36	38	31	55	55	100						
AtCAX1	64	62	57	48	49	38	100					
AtCAX2	46	49	44	72	72	54	46	100				
AtCAX3	67	66	56	50	48	39	79	48	100			
AtCAX4	56	59	49	46	47	34	57	47	59	100		
AtCAX5	48	49	44	72	73	52	48	87	49	47	100	
AtCAX6	47	45	38	66	65	53	46	82	47	45	88	

## Data Availability

All data generated or analyzed during this study are included in this published article.

## Supplementary Materials

The following are available online at https://www.mdpi.com/article/10.3390/ijms22158186/s1.

## Abbreviations

ABC	ATP-binding cassette
CAX	Cation/H^+^ exchanger
CaCA	Ca^2+^/cation antiporter
CCX	Cation/Ca^2+^ exchanger
CDT	Cation diffusion facilitator
HMA	Heavy metal ATPase
ICP-MS	Inductively coupled plasma mass spectrometry
MAPK	Mitogen-activated protein kinase
MHX	Mg^2+^/H^+^ exchanger
MTP	Metal tolerance protein
NRR	N-terminal regulatory region
TF	Transcription factors
TMDs	Transmembrane domains
