# Supplementary material for: The Rice Cation/H+ Exchanger Family Involved in Cd Tolerance and Transport
Source: Int J Mol Sci. 2021 Jul 30;22(15):8186. doi: 10.3390/ijms22158186 (PMC8348036; doi:10.3390/ijms22158186)
Supplement: Supplementary file 1 [file ijms-22-08186-s001.zip › ijms-1287743-supplementary.pdf]

Supplementary Material

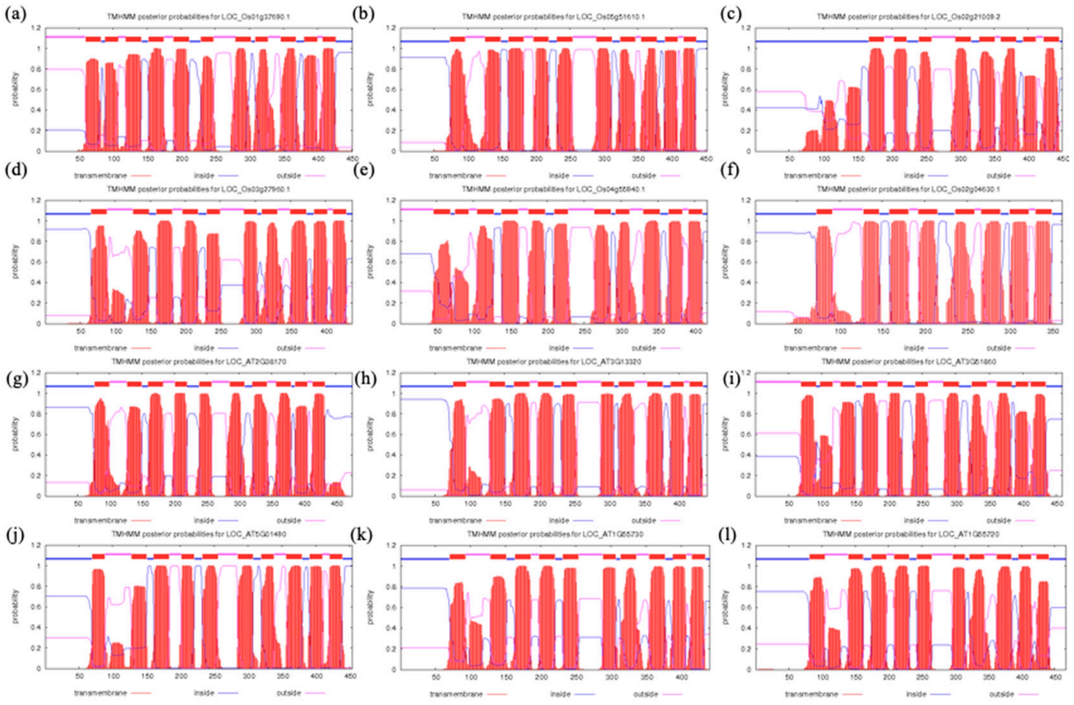

Figure 1. Membrane topology of selected CAXs. The topologic models were generated by the TMHMM program (www.cbs.dtu.dk/services/TMHMM/). TMHMM posterior probabilities for (a) OsCAX1a; (b) OsCAX1b; (c) OsCAX1c; (d) OsCAX2; (e) OsCAX3; (f) OsCAX4; (g) AtCAX1; (h) AtCAX2; (i) AtCAX3; (j) AtCAX4; (k) AtCAX5; (l) AtCAX6. The red areas indicate the predicted TM domains, and the blue and magenta lines indicate the predicted regions inside or outside of the membrane, respectively.

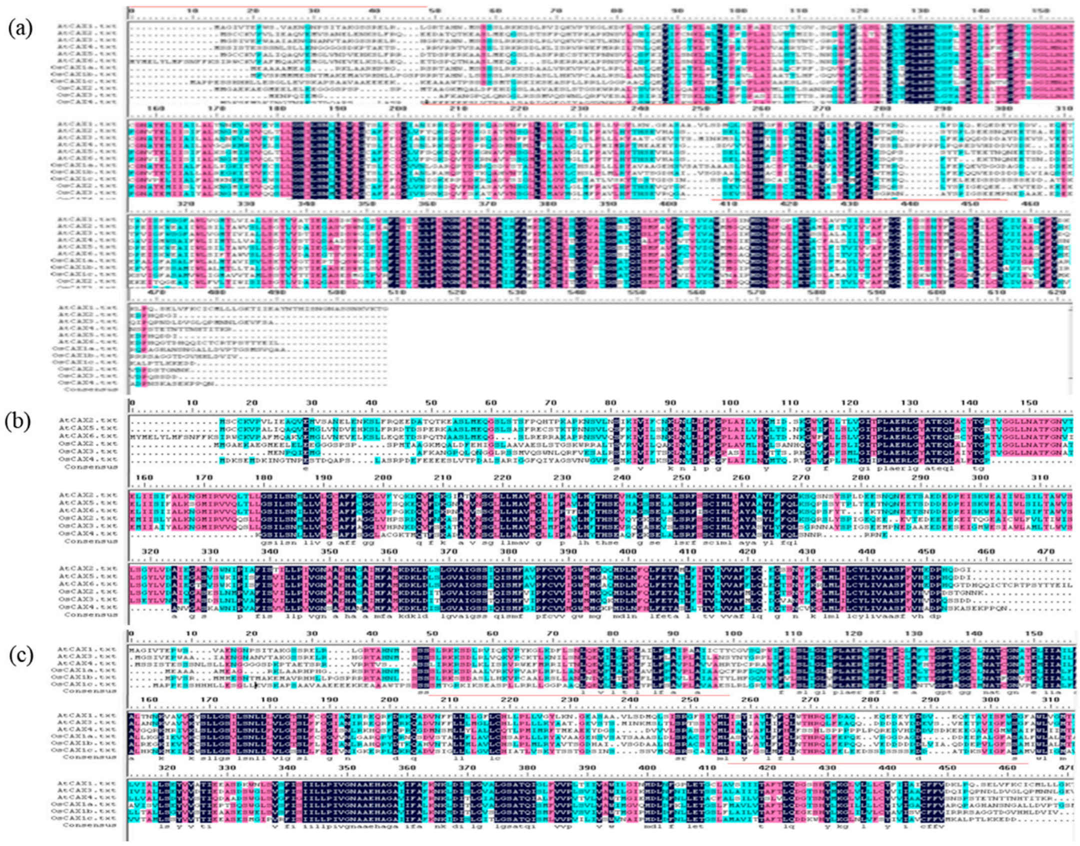

Figure S2. Multiple alignment of *Oryza sativa* and *Arabidopsis thaliana* CAX family proteins. (a) Amino acid sequence alignments; (b) the subfamily IB; (c) the subfamily IA. Multiple alignment was performed with DNAMAN. Black, red and blue lines underneath alignments indicate reported consensus transport residues more frequently reported consensus transport residues, and less frequently reported consensus transport residues, respectively. "|" above the sequence mean every ten amino acid residues.

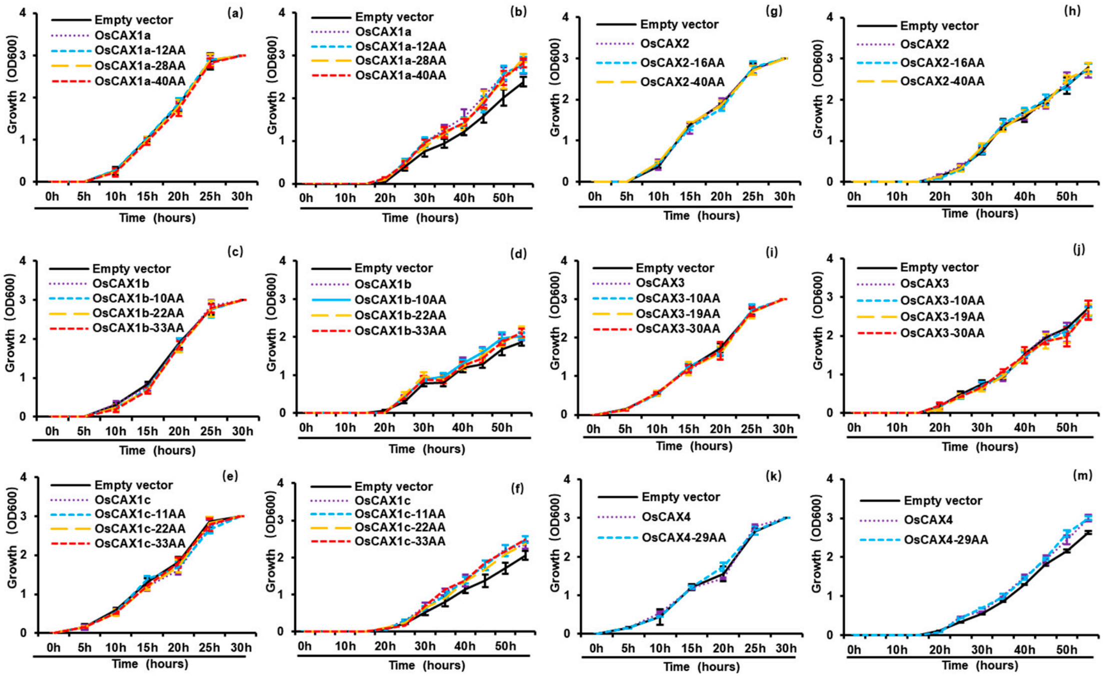

Figure S3. Functional assay of rice *CAX* genes by heterologous expression in yeast. Yeast strains were grown in liquid media with 0 μmol/L $CdCl_2$ for 30 h (a), (c), (e), (g), (i) and (k); or 10 μmol/L $CdCl_2$ for 55 h (b), (d), (f), (h), (j) and (m). The absorbance at 600 nm (OD600) of cell cultures was measured every 5 h. Values are mean ± SE (n = 3).

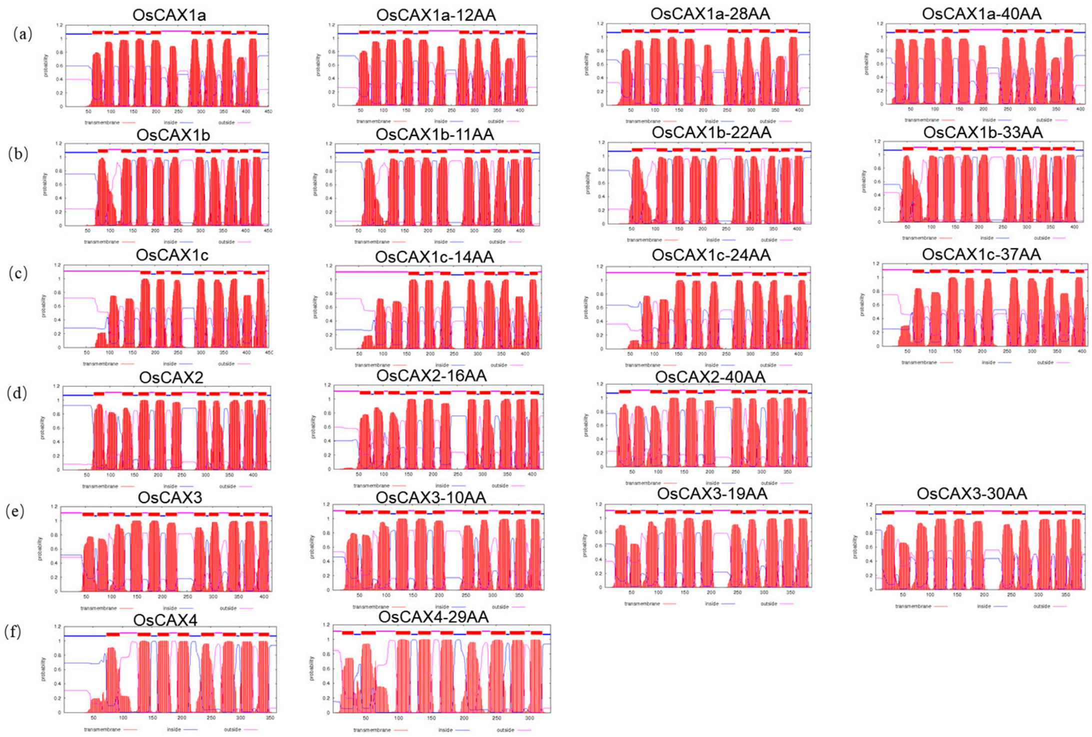

Figure S4. Transmembrane structure of the truncated rice CAX proteins. The topologic models were generated by the TMHMM program. TMHMM posterior probabilities for (a) OsCAX1a, OsCAX1a-12A, OsCAX1a-28AA, OsCAX1a-40AA; (b) OsCAX1b, OsCAX1b-11AA, OsCAX1b-22AA, OsCAX1b-33AA; (c) OsCAX1c-14AA, OsCAX1c-24AA, OsCAX1c-37AA; (d) OsCAX2, OsCAX2-16AA, OsCAX2-40AA; (e) OsCAX3, OsCAX3-10AA, OsCAX3-19AA, OsCAX3-40AA; (f) OsCAX4, OsCAX4-29AA. The red, blue and magenta areas indicate the predicted TM domains, the regions inside or outside of the membrane, respectively.

Table S1. Primers for qRT-PCR analysis of rice *CAX* family genes.

Genes	Forward primer (5'-3')	Reverse primer (5'-3')
Osactin	GGAACTGGTATGGTCAAGGC	AGTCTCATGGATAACCGCAG
OsCAX1a	TGTGGGTGTTCGCTCTTAGT	GTGCAATCTGCTCTGTGAGG
OsCAX1b	ATCTCAACGACGTCCTCCTC	GCTAAGCACAAACACCCACA
OsCAX1c	TGAGCTTCCTGAGCGAACAT	TTATGCAGAGCGAACAGTGC
OsCAX2	CGTCATGCTCCACTACCTCT	TCCCAATCTCTCAGCCAAGG
OsCAX3	CTGGCGGTATTGTTCATCGG	AGTCCCATGACAGCCATCAA
OsCAX4	TTCCTTTGGCTGAGCGATTG	ACTCGGTTGCATTGCCAAAT

Table S2. Primers used to amplify the open reading frame of the candidate genes.

Genes	Primer (5'-3')
OsCAX1a-F	ATGGAGGCGG CGGCGGCGAT
OsCAX1a-14AA-F	ATGGCGGCGCGGCACCCGCACGG
OsCAX1a-28AA-F	ATGTCGTCGTCGCTGCGGAAGAA
OsCAX1a-40AA-F	ATGGTGCGGAAGGTCCCCGTGGC
OsCAX1a-R	TCATGCGGCCTGAACACTCAT
OsCAX1b-F	ATGCCAGTGT CGCGGATGAT GAT
OsCAX1b-10AA-F	ATGACGATGGCCAAGGAGATGGC
OsCAX1b-22A-F	ATGCTTCCGGGGTCTCCTCGCCG
OsCAX1b-33A-F	ATGAACCTCTCATCTTCCTCCCT
OsCAX1b-R	CTACACAATC ACATCCAGATGG
OsCAX1c-F	ATGGCGCCGCCGGAGAGCA
OsCAX1c-14AA-F	ATGCTGCTTGAAGTCAGCAAGGC
OsCAX1c-24AA-F	ATGGCGGTGGCTGCCGAGGAAGA
OsCAX1c-37AA-F	ATGGCGTGGACACCGTCGTCGTC
OsCAX1c-R	TTAGTCATCTTCCTTTTTTAGCGTTGGG
OsOsCAX2-F	ATGATGGGGGCGGAGAAGG
OsCAX2-14AA-F	ATGGAGGAGGGCGGGGGCT
OsCAX2-40AA-F	ATGGGGTCGCTCGCCGCGGT
OsCAX2-R	TCACTTGTTGTTACCTGT
OsCAX3-F	ATGGAGAATCCTCAGATTG
OsCAX3-10AA-F	ATGGGTGGGCTACGTTCCAGCAT
OsCAX3-19AA-F	ATGGTTCAGTCCTGGAACCTGCA
OsCAX3-30AA-F	ATGAACCTGCAGAGATTCGTTGA
OsCAX3-R	TCAATCATC CTGGATTGTG G
OsCAX4-F	ATGAGCCAACAGCTTCCAA
OsCAX4-29AA-F	ATGCAGGCCCCTTCATTGGCTAG
OsCAX4-R	TTAGTTTTGTGGAGGCTTCT

Table S3. The *CAX* family genes involve in metal stress tolerance.

Genes	Species	Subcellular localization	Functions	References
OsCAX1a	Yeast	Vacuolar	Ca transport and Ca homeostasis	[1, 2]
OsCAX1b	Yeast	-	Ca tolerance	[1]
OsCAX1c	Yeast	-	Ca tolerance	[1]
OsCAX3	Yeast	Plasma membrane	Ca tolerance	[1]
OsCAX4	Yeast	-	Ca, Cu and Mn transport	[3]
AtCAX1	Yeast	-	Ca, Mn and Ba tolerance; Ca, Zn and Cd transport	[4-6]
	Arabidopsis	Vacuolar	Ca, Mn, Mg, salt and freezing tolerance; Ca transport	[7-9]
	Arabidopsis halleri	-	Cd tolerance	[10, 11]
	Tobacco	-	Ca homeostasis	[12]
	Petunia	-	Cd tolerance and accumulation	[13]
	Tomato	-	Ca accumulation	[14]
AtCAX2	Yeast	Vacuolar	Ca tolerance and translocation	[15-17]
	Tobacco	-	Cd, Mn, Zn tolerance; Cd and Mn transport	[18-21]
	Potato	-	Ca transport	[22]
	Tomato	-	Ca accumulation	[23]
AtCAX3	Yeast	Vacuolar	Ca tolerance and translocation	[24]
	Arabidopsis	-	salt tolerance	[25]
AtCAX4	yeast	Vacuolar	Ca, Ba and salt tolerance	[26]
	Arabidopsis	-	Cd, Ca and salt tolerance	[9, 27]
	Tobacco	-	Cd, Mn and Zn tolerance; Cd transport and accumulation	[19-21]
	tomato	-	Ca accumulation	[28]
AtCAX5	Yeast	Vacuolar	Ca and Mn transport; ion homeostasis	[16, 29]
PutCAX1	Yeast	Vacuolar	Ca and Ba tolerance	[6]
PutCAX2	Yeast	Golgi apparatus	Ca and Ba tolerance	[26]
TuCAX1a	Arabidopsis	-	Ca and Zn tolerance and translocation	[30]
TuCAX1b	Arabidopsis	-	Ca and Zn tolerance; Cd, Ca and Zn translocation	[30]
LeCAX2	Yeast	-	Ca and Mn transport	[16]
GhCAX3	Yeast	-	Ca Transport and stress tolerance	[31]
GhCAX3	Arabidopsis	-	cross-talk of ABA and cold signal transduction	[31]
SeCAX3	Yeast	-	Ca transport	[32]
VvCAX3	Yeast	-	Na, Li and Cu tolerance; Ca transport	[33]

SaCAX2	Sedum alfredii	-	Ca and Mn sequestration; Cd accumulation	[32]

References

1. Kamiya, T.; Akahori, T.; Maeshima, M. Expression profile of the genes for rice cation/H^+ exchanger family and functional analysis in yeast. *Plant Cell Physiol*. **2005**, *46*, 1735–1740.
2. Kamiya, T.; Akahori, T.; Ashikari, M.; Maeshima, M. Expression of the vacuolar Ca^{2+}/H^+ exchanger, OsCAX1a, in rice: cell and age specificity of expression, and enhancement by Ca^{2+}. *Plant Cell Physiol*. **2006**, *47*, 96–106.
3. Yamada, N.; Theerawitaya, C.; Cha-um, S.; Kirdmanee, C.; Takabe, T. Expression and functional analysis of putative vacuolar Ca^{2+}-transporters (CAXs and ACAs) in roots of salt tolerant and sensitive rice cultivars. *Protoplasma* **2014**, *251*, 1067–1075.
4. Shigaki, T.; Barkla, B.J.; Miranda-Vergara, M.C.; Zhao, J.; Pantoja, O.; Hirschi, K.D. Identification of a crucial histidine involved in metal transport activity in the Arabidopsis cation/H^+ exchanger CAX1. *J. Biol. Chem*. **2005**, *280*, 30136–30142.
5. Shigaki. T.; Mei, H.; Marshall, J.; Li, X.; Manohar, M.; Hirschi, K.D. The expression of the open reading frame of Arabidopsis *CAX1*, but not its cDNA, confers metal tolerance in yeast. *Plant Biol. (Stuttg)*. **2010**, *12*, 935–939.
6. Guo, H.; Tsugama, D.; Takano, T.; Bu, Y.; Liu, S. AtCAX1 N-terminal can improve Ca^{2+} and Ba^{2+} tolerance in yeast. *Cell Biol. Biophys*. **2015**, *4*, 1–4.
7. Catala, R.; Santos, E.; Alonso, J.M.; Ecker, J.R.; Martinez-Zapater, J.M.; Salinas, J. Mutations in the Ca^{2+}/H^+ transporter CAX1 increase *CBF*/*DREB1* expression and the cold-acclimation response in Arabidopsis. *Plant Cell*. **2003**, *15*, 2940–2951.
8. Cheng, N.H.; Pittman, J.K.; Barkla, B.J.; Shigaki, T.; Hirschi, K.D. The Arabidopsis *cax1* mutant exhibits impaired ion homeostasis, development, and hormonal responses and reveals interplay among vacuolar transporters. *Plant Cell*. **2003**, *15*, 347–364.
9. Bu, Y.; Fu, W.; Chen, J.; Takano, T.; Liu, S. Description of *AtCAX4* in Response to Abiotic Stress in Arabidopsis. *Int. J. Mol. Sci*. **2021**, *22*, 856.
10. Baliardini, C.; Meyer, C.L.; Salis, P.; Saumitou-Laprade, P.; Verbruggen, N. CATION EXCHANGER1 Cosegregates with Cadmium Tolerance in the Metal Hyperaccumulator Arabidopsis halleri and Plays a Role in Limiting Oxidative Stress in Arabidopsis Spp. *Plant Physiol*. **2015**, *169*, 549–559.
11. Ahmadi, H.; Corso, M.; Weber, M.; Verbruggen, N.; Clemens, S. *CAX1* suppresses Cd-induced generation of reactive oxygen species in *Arabidopsis halleri*. *Plant Cell Environ*. **2018**, *41*, 2435–2448.
12. Hirschi, K. Expression of Arabidopsis *CAX1* in tobacco: altered calcium homeostasis and increased stress sensitivity. *Plant Cell*. **1999**, *11*, 2113–2122.
13. Wu, Q.; Shigaki, T.; Williams, K.A.; Han, J.S.; Kim, C.K.; Hirschi, K.D.; Park, S. Expression of an Arabidopsis Ca^{2+}/H^+ antiporter CAX1 variant in petunia enhances cadmium tolerance and accumulation. *J. Plant Physiol*. **2011**, *168*, 167–173.
14. Zorrilla, C.; Schabow, J.E.; Chernov, V.; Plata, J.P. CAX1 Vacuolar Antiporter Overexpression in Potato Results in Calcium Deficiency in Leaves and Tubers by Sequestering Calcium as Calcium Oxalate. *Crop Sci*. **2018**, *59*, 1.
15. Hirschi. K.D.; Zhen, R.G.; Cunningham, K.W.; Rea, P.A.; Fink, G.R. CAX1, an H^+/Ca^{2+} antiporter from Arabidopsis. *Proc. Natl. Acad. Sci. USA*. **1996**, *93*, 8782–8786.
16 Edmond, C.; Shigaki, T.; Ewert, S.; Nelson, M.D.; Connorton, J.M.; Chalova, V.; Noordally, Z.; Pittman, J.K. Comparative analysis of CAX2-like cation transporters indicates functional and regulatory diversity. *Biochem. J*. **2009**, *418*, 145–154.
17. Pittman, J.K.; Shigaki, T.; Hirschi, K.D. Evidence of differential pH regulation of the Arabidopsis vacuolar $Ca2^+$/H^+ antiporters CAX1 and CAX2. *FEBS Lett*. **2005**, *579*, 2648–2656.
18. Hirschi, K.D.; Korenkov, V.D.; Wilganowski, N.L.; Wagner, G.J. Expression of arabidopsis *CAX2* in tobacco. Altered metal accumulation and increased manganese tolerance. *Plant Physiol*. **2000**, *124*, 125–133.
19. Koren'kov, V.; Park, S.; Cheng, N.H.; Sreevidya. C.; Lachmansingh, J.; Morris, J.; Hirschi, K.; Wagner, G.J. Enhanced Cd^{2+}-selective root-tonoplast-transport in tobaccos expressing Arabidopsis cation exchangers. *Planta*. **2007**, *225*, 403–411.
20. Korenkov, V.; Hirschi, K.; Crutchfield, J.D.; Wagner, G.J. Enhancing tonoplast Cd/H antiport activity increases Cd, Zn, and Mn tolerance, and impacts root/shoot Cd partitioning in *Nicotiana tabacum* L. *Planta* **2007**, *226*, 1379–1387.
21. Korenkov, V.; King, B.; Hirschi, K.; Wagner, G.J. Root-selective expression of *AtCAX4* and *AtCAX2* results in reduced lamina cadmium in field-grown *Nicotiana tabacum* L. *Plant Biotechnol. J*. **2009**, *7*,

219–226.
22. Kim, C.K.; Han, J.S.; Lee, H.S.; Oh, J.Y.; Shigaki, T.; Park, S.H.; Hirschi, K. Expression of an Arabidopsis *CAX2* variant in potato tubers increases calcium levels with no accumulation of manganese. *Plant Cell Rep*. **2006**, *25*, 1226–1232.
23. Mi, Y.C.; Han, J.S.; Giovannoni, J.; Yang, L.; Chang, K.K.; Lim, K.B.; Chung, J.D. Modest calcium increase in tomatoes expressing a variant of Arabidopsis cation/H^+ antiporter. *Plant Biotechnol. Rep*. **2010**, *4*, 15–21.
24. Shigaki, T.; Cheng, NH.; Pittman, J.K.; Hirschi, K. Structural determinants of Ca^{2+} transport in the Arabidopsis H^+/Ca^{2+} antiporter CAX1. *J. Biol. Chem*. **2001**, *276*, 43152–43159.
25. Zhao, J.; Barkla, B.J.; Marshall, J.; Pittman, J.K.; Hirschi, K.D. The Arabidopsis *cax3* mutants display altered salt tolerance, pH sensitivity and reduced plasma membrane H^+-ATPase activity. *Planta* **2008**, *227*, 659–669.
26. Chen, J.; Takano, T.; Liu, S.; Bu, Y. Identification and characterization of a $cation^{2+}/H^+$ antiporter Atcax4 gene from *Arabidopsis thaliana*. *Mol. Soil. Biol*. **2016**, *7*, 1–5.
27. Liao, Q.; Jian, S.F.; Song, H.X.; Guan, C.Y.; Lepo, J.; Ismail, A.M.; Zhang, Z.H. Balance between nitrogen use efficiency and cadmium tolerance in *Brassica napus* and *Arabidopsis thaliana*. *Plant Sci*. **2019**, *284*, 57–66.
28. Jeong, S.W.; Han, J.S.; Kim, K.M.; Oh, J.Y.; Chung, J.D. Expression of Arabidiopsis *CAX4* in tomato fruits increases calcium level with no accumulation of other metallic cations. *J. Plant Biotechnol*. **2008**, *35*, 337–343.
29. Choe, M.; Choe, W.; Cha, S.; Lee, I. Changes of cationic transport in AtCAX5 transformant yeast by electromagnetic field environments. *J. Biol. Phys*. **2018**, *44*, 433–448.
30. Qiao, K.; Wang, F.; Liang, S.; Hu, Z.; Chai, T. Heterologous expression of *TuCAX1a* and *TuCAX1b* enhances Ca^{2+} and Zn^{2+} translocation in Arabidopsis. *Plant Cell Rep*. **2019**, *38*, 597–607.
31. Xu. L.; Zahid, K.R.; He, L.; Zhang, W.; He, X.; Zhang, X.; Yang, X.; Zhu, L. GhCAX3 gene, a novel Ca^{2+}/H^+ exchanger from *cotton*, confers regulation of cold response and ABA induced signal transduction. *PLoS ONE* **2013**, *8*, e66303.
32. Zhang, L.; Hao, J.; Bao, M.; Hasi, A.; Niu, Y. Cloning and characterization of a Ca^{2+}/H^+ exchanger from the halophyte *Salicornia europaea* L. *Plant Physiol. Biochem*. **2015**, *96*, 321–328.
33. Martins, V.; Carneiro, F.; Conde, C.; Sottomay, M.; Geros, H. The grapevine VvCAX3 is a cation/$H(^+)$ exchanger involved in vacuolar $Ca(^{2+})$ homeostasis. *Planta* **2017**, *246*, 1083–1096.
